# Survival Analysis after Living Donor Liver Transplantation for Hepatocellular Carcinoma: A Single Center Cohort Study

**DOI:** 10.3390/biology10050446

**Published:** 2021-05-20

**Authors:** Byung-Gon Na, Seong-Hoon Kim, Sang-Jae Park

**Affiliations:** Organ Transplantation Center, National Cancer Center, 323 Ilsan-ro, Ilsandong-gu, Goyang-si 10408, Gyeonggi-do, Korea; hbpbgna@gmail.com (B.-G.N.); spark@ncc.re.kr (S.-J.P.)

**Keywords:** hepatocellular carcinoma, living donor, liver transplantation, survival analysis

## Abstract

**Simple Summary:**

Patients with hepatocellular carcinoma (HCC) exceeding the Milan criteria and an immediate pretransplant AFP > 400 ng/mL had unfavorable survival outcomes following living-donor liver transplantation. The tumor size, poorly histologic differentiation, and the presence of microvascular invasion of HCC in the explanted liver were independent risk factors for both overall survival and recurrence-free survival.

**Abstract:**

Background: Living-donor liver transplantation (LDLT) for hepatocellular carcinoma (HCC) has been used as a curative treatment option for hepatocellular carcinoma (HCC) because of a shortage of deceased donors. This study aimed to investigate survival outcomes after LDLT for HCC. Method: This study included 359 patients undergoing LDLT for HCC. We analyzed overall survival (OS) and recurrence-free survival (RFS) and the prognostic factors related to them. Results: The 5-year OS and RFS rates of patients within the Milan criteria (WM) were better than those of patients beyond the Milan criteria (BM) (87.3% vs. 64.1% and 87.6% vs. 57.8%, respectively, both *p* < 0.05). Alpha-fetoprotein level (AFP) > 400 ng/mL (hazard ratio (HR), 2.07; 95% CI, 1.28–3.36; *p <* 0.05) and HCC of BM (HR, 2.61; 95% CI, 1.60–4.26; *p <* 0.05) at immediate pretransplant were independent risk factors of OS. AFP > 400 ng/mL (HR, 2.16; 95% CI, 1.34–3.49; *p* < 0.05) and HCC of BM (HR, 3.01; 95% CI, 1.81–5.01; *p* < 0.05) were also independent risk factors of RFS. In pathologic findings of explanted liver, tumor size, Edmondson–Steiner grade III–IV, and microvascular invasion were independent risk factors of both OS and RFS (*p* < 0.05). Conclusions: BM and AFP > 400 ng/mL at immediate pretransplant are unfavorable predictors of survival outcomes after LDLT for HCC.

## 1. Introduction

Hepatocellular carcinoma (HCC) accounts for most primary liver cancer. Primary liver cancer is the sixth most commonly diagnosed cancer and the second most common cause of death worldwide [1]. Upon diagnosis, patients with HCC must be evaluated to assess tumor burden and liver function before suitable treatment can be established. Liver resection is considered primarily for solitary tumors in patients with normal portal pressure and bilirubin levels [2]. Liver transplantation (LT) is a radical treatment for tumors and underlying liver disease, especially in patients with cirrhotic livers.

According to a 2016 report on Korean organ transplantation, the number of living-donor liver transplantations (LDLT) is increasing, and presently accounts for approximately 65% of LT cases. When indications for LDLT are reviewed, liver cirrhosis with hepatitis B virus accounts for most LDLT cases, followed by malignant neoplasms. Deceased-donor liver transplantation (DDLT) is usually not an option for patients with HCC because of donor shortage. Thus, LDLT performs a pivotal role in the treatment of patients with HCC.

The Milan criteria (solitary tumor ≤5 cm in size or ≤3 tumors each with a size <3 cm) were suggested based on the finding that patients with HCC within the criteria had better survival outcomes than those beyond the criteria [3]. These criteria have been adopted for the selection of patients who will benefit with regard to survival outcomes after DDLT. Compared to DDLT, patient selection in LDLT is not dependent on organ allocation systems and hence more liberal, expanded criteria can be applied. Therefore, considering that the current situation shows an increase in LDLT for HCC, we retrospectively analyzed the overall survival (OS) and recurrence-free survival (RFS) and sought to find risk factors associated with survival outcomes after LDLT.

## 2. Methods

### 2.1. Study Patient Selection

**Figure 1:**
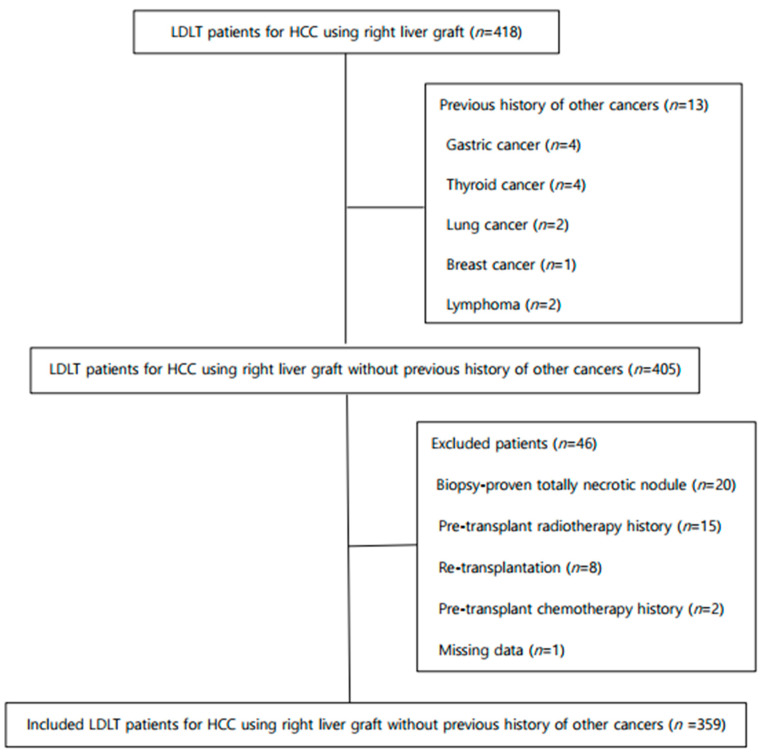
Study flowchart. Patients undergoing LDLT for HCC without the history of other cancers. HCC, hepatocellular carcinoma; LDLT, living-donor liver transplantation.

Four-hundred thirty-five patients with HCC underwent LT from January 2005 to December 2015. Of these patients, 16 patients underwent DDLT and one patient received an extended left liver graft. Fifty-nine of the remaining 418 patients were excluded according to the protocol described in Figure 1. A total of 359 patients were eventually included in this study. Data were retrospectively collected from the LT database at our institution. This study was approved by the Institutional Review Board according to the recommendations of the Declaration of Helsinki (approval number: NCC2018-0032 and date of approval 22 November 2018). The need for informed consent was waived.

### 2.2. Pretransplant Imaging Work-Up for Recipient

Our policy for selecting patients with HCC for LDLT was based on preoperative imaging tools such as computed tomography (CT) and magnetic resonance imaging (MRI) of the liver. A chest CT, bone scan, and ^18^Fluoro-2-deoxy-D-glucose positron emission tomography/computed tomography (^18^F-FDG PET/CT) were performed to confirm the absence of extrahepatic metastasis of HCC. We did not perform the biopsy for preoperative tumor pathology because of the possibility of needle tract tumor seeding.

### 2.3. Living Donor Protocol

Living donors were volunteers who provided signed informed consent with the approval of the Korean Network for Organ Sharing after undergoing full medical and psychiatric assessments by healthcare professionals. Imaging evaluations included Doppler ultrasonography (US), triphasic CT with liver volumetry, and magnetic resonance cholangiography. In principle, a graft-to-recipient weight ratio (GRWR) of 0.8% was chosen as the minimum cut-off value for recipients [4]. The selection criteria and operative procedures of living donor hepatectomy are described in previous studies [5,6].

### 2.4. Recipient Liver Transplantation

After total hepatectomy of the liver with HCC, graft implantation was initiated with a right hepatic vein anastomosis to the inferior vena cava. The right portal vein of the graft was anastomosed to the recipient’s right or main portal vein followed by graft reperfusion. The right hepatic artery of the graft was anastomosed with the right or left hepatic artery of the recipient under microscopic guidance. Biliary reconstruction was performed in a duct-to-duct fashion.

### 2.5. Postoperative Complications, Follow-Up, and Surveillance

Postoperative complications within 30 days after LDLT were assessed using the Clavien–Dindo (C–D) classification. Moderate or severe complications were defined as ≥ C–D grade III. HCC recurrence was defined based on periodic radiological examinations during the follow-up period. Patient serum alpha-fetoprotein (AFP) levels were measured every 1–2 months, and chest radiography and abdominal US or CT were performed every 3–6 months. MRI could be performed for further evaluation.

### 2.6. Statistical Analysis

Categorical variables are shown as frequencies and percentages. Continuous variables are expressed as means ± standard deviations (SDs) or median (interquartile range, IQR) according to the normality assumption. Chi-square and Fisher’s exact tests were used to analyze categorical variables, whereas Student’s t-tests and Mann–Whitney U-tests were applied for continuous variables. The RFS and OS rates were estimated using the Kaplan–Meier method, and the survival curves were compared using the log-rank test. The survival endpoint for RFS was the time of HCC recurrence, which was defined as the time when CT imaging initially detected HCC recurrence. In this study, survival, follow-up loss, patient’s death, and occurrence of other cancer except HCC were censored. We also tried to consider disease-free survival, but only focused on the RFS of HCC in this study. The optimal AFP cut-off value (400 ng/mL) was determined based on previous studies in which the immediate pretransplant AFP > 400 ng/mL was identified as a risk factor for HCC recurrence after LT [7,8]. The logistic regression model was used for univariate and multivariate analyses for risk factor of recurrence. The Cox proportional hazards model was used to identify the prognostic factors associated with RFS and OS. Variables included in the multivariable Cox proportional hazards model were selected from the univariate results. *p* value < 0.05 was considered statistically significant. All analyses were performed using computer software SPSS Version 20.0 (SPSS Inc., Chicago, IL, USA).

## 3. Results

### 3.1. Baseline Characteristics

Table 1 summarizes the baseline characteristics of the recipients and living donors. Among the included patients, 192 (53.5%) fulfilled the Milan criteria (within the Milan, WM) and 167 (46.5%) exceeded the criteria (beyond the Milan, BM). Both groups exhibited similar characteristics. However, there were significant differences in the proportion of male patients (*p* < 0.05), AFP level (*p* < 0.05), and the number of patients treated using transcatheter arterial chemoembolization (*p* < 0.05).

### 3.2. Association of Milan Criteria with Explanted Liver Pathology

Table 2 shows the difference between the pathologic findings of explanted livers from the two groups. The BM group was statistically more associated with Edmondson–Steiner (E–S) histologic grade III-IV tumors (odds ratio (OR), 2.62; 95% confidence interval (CI), 1.67–4.11; *p* < 0.05), microvascular invasion (OR, 3.8; 95% CI, 2.42–5.96; *p* < 0.05), serosal invasion (OR, 3.69; 95% CI, 2.13–6.39; *p* < 0.05), and intrahepatic metastasis (OR, 7.22; 95% CI, 4.49–11.62; *p* < 0.05), compared to the WM group.

### 3.3. Postoperative Complications

For all LDLT recipients, the median length of hospital stay was 15 days (13–18 days) and operation time was 420 min (357–497 min). Postoperative complications developed in 126 recipients (35.1%). Moderate and severe complications ≥ C–D grade III consisted of biliary leakage and stricture in 83 patients (23.1%), postoperative bleeding in 15 (3.7%), pneumonia in eight (2.2%) exacerbated to sepsis, hepatic artery thrombosis (HAT) in seven (1.7%), and uncontrolled pleural effusion in an attempt to drain one patient. The recipients with biliary complications were managed with radiologic or endoscopic interventions. Postoperative bleeding required immediate exploration, and HAT was resolved with percutaneous transluminal angioplasty such as thrombolysis and stents when the artery was kinked and twisted in angiographic findings. The remaining complications <grade III including pleural effusion, ileus, and wound dehiscence were treated without any surgical intervention.

### 3.4. Survival Outcomes after LDLT for HCC

The median follow-up period for patients after LDLT was 55.8 months. The 1-, 3-, and 5-year OS rates for all included patients were 91.9%, 82.2%, and 76.4%, respectively, whereas the 1-, 3-, and 5-year HCC RFS rates were 83.2%, 76.9%, and 73.8%, respectively. Of the 88 (24.5%) patients who died after LDLT, 61 (16.9%) died from HCC recurrence and the others died from non-HCC-related causes, such as graft failure (*n* = 13, 3.6%), sepsis (*n* = 8, 2.2%), and other causes (*n* = 7, 1.9%) including de-novo cancers (*n* = 4), brain hypoxic injury (*n* = 1), and unknown (*n* = 1). First, the causes of graft failure included biliary cirrhosis in four patients with recurrent biliary stricture, chronic rejection in three with recurrent acute cellular rejection, liver failure in three with HCV recurrence, and alcoholism in three patients with a previous alcoholic history. Four patients died from de-novo cancers such as advanced gastric cancer, esophageal cancer, mantle cell lymphoma, and lymphoplasmacytic lymphoma. One patient underwent percutaneous drainage for uncontrolled pleural effusion, and unfortunately, developed hypoxic brain damage due to injury to the pulmonary artery during the procedure, which required mechanical ventilation for two years. A case of unknown cause of death happened after a sudden cardiac arrest on the 7th postoperative day in the ward, and spontaneous circulation was not restored despite cardiopulmonary resuscitation.

Of the 91 (25.34%) patients with recurrent HCC during the follow-up period, intrahepatic, extrahepatic, and simultaneous recurrence at both sites occurred in 20 (5.5%), 67 (18.6%), and 4 (1.1%) patients, respectively. The sites of extrahepatic recurrence included 33 cases in the lung, 14 in bone, eight in intra-peritoneal seeding nodules, six in intra-abdominal lymph nodes, two in the adrenal glands, two in the brain, and two in the diaphragm. The recurred HCC after LDLT was treated with curative intent whenever possible, otherwise with palliative intent. Local treatments such as transarterial chemoembolization (TACE), radiofrequency ablation (RFA), and radiation therapy were chosen in cases of intrahepatic recurrence, whereas surgical resection, chemotherapy, radiation therapy, and systemic therapy with a tyrosine-kinase inhibitor could be attempted for extrahepatic recurrence. The median follow-up period for patients with HCC recurrence was 34.1 (IQR, 22–45.8) months.

**Figure 2:**
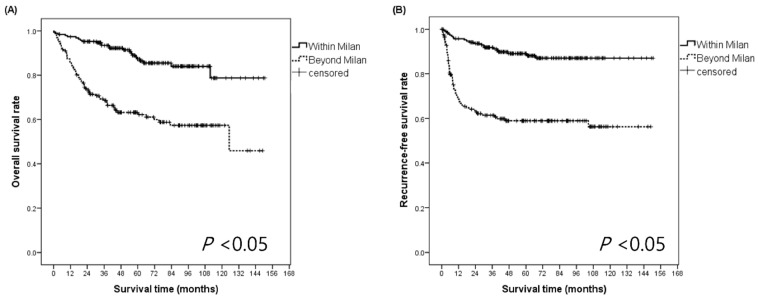
Comparison of the overall survival rates and recurrence-free survival rates for patients with HCC assessed according to the Milan criteria. (**A**) The overall survival rates at 1, 3, and 5 years for WM patients were 97.4%, 93.5%, and 87.3%, respectively; those for BM patients were 85.6%, 69.4%, and 64.1%, respectively (**B**) The recurrence-free survival rates at 1, 3, and 5 years for patients within the Milan criteria were 95.7%, 90.4%, and 87.6%, respectively; those for patients beyond the Milan criteria were 68.6%, 61.2%, and 57.8%, respectively. BM, beyond the Milan criteria; HCC, hepatocellular carcinoma; LDLT, living-donor liver transplantation; WM, within the Milan criteria.

Post-LDLT survival analyses were performed for the WM and BM groups (Figure 2A,B). The 1-, 3-, and 5-year OS rates for the WM patients were 97.4%, 93.5%, and 87.3%, whereas those for the BM patients were 85.6%, 69.4%, and 64.1%, respectively (*p* < 0.05). The 1-, 3-, and 5-year RFS rates for the WM patients were 95.7%, 90.4%, and 87.6%, whereas those for the BM patients were 68.6%, 61.2%, and 57.8%, respectively (*p* < 0.05).

### 3.5. Prognostic Factors Related to OS and RFS

We conducted univariate and multivariate analyses to assess the clinical and pathological factors that may influence the OS and RFS prognoses for all included patients. In multivariate analyses for OS, AFP > 400 ng/mL (hazard ratio (HR), 2.07; 95% CI, 1.28–3.36; *p <* 0.05) and HCC of BM (HR, 2.61; 95% CI, 1.60–4.26; *p <* 0.05) were independent risk factors for OS among the preoperative characteristics. Furthermore, the greatest tumor size (HR, 1.15; 95% CI, 1.07–1.23; *p <* 0.05), E–S histologic grade III–IV (HR, 2.06; 95% CI, 1.34–3.18; *p* < 0.05), and presence of microvascular invasion (HR, 2.39; 95% CI, 1.32–4.35; *p* < 0.05) were independently unfavorable factors for OS (Table 3). Regarding RFS, the following variables were identified as independent risk factors in the univariate analysis (*p <* 0.05): model for end-stage liver disease; AFP; the Milan criteria; number of tumors; tumor size; E–S histologic grade; and the presence of microvascular invasion, bile duct invasion, serosal invasion, and intrahepatic metastasis. In multivariate analysis for RFS, AFP > 400 ng/mL (HR, 2.16; 95% CI, 1.34–3.49; *p* < 0.05), HCC of BM (HR, 3.01; 95% CI, 1.81–5.01; *p* < 0.05), the greatest tumor size (HR, 1.24; 95% CI, 1.16–1.33; *p* < 0.05), E–S histologic grade III–IV (HR, 2.25; 95% CI, 1.45–3.49; *p* < 0.05), and presence of microvascular invasion (HR, 2.15; 95% CI, 1.21–3.82; *p* < 0.05) were identified as independent risk factors for unfavorable RFS (Table 3).

### 3.6. RFS Analysis According to AFP Stratified by the Milan Criteria

**Figure 3:**
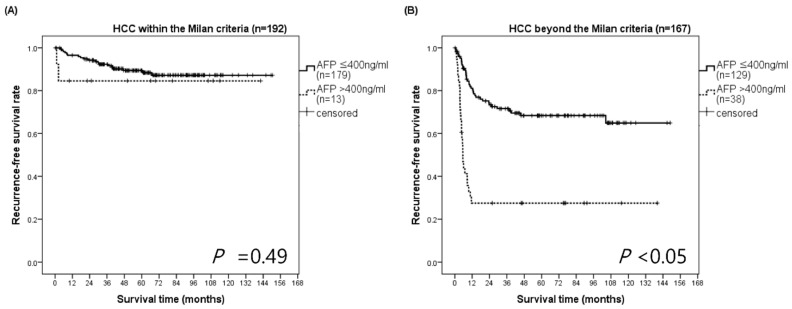
Recurrence-free survival according to the Milan criteria in the subgroups stratified by AFP. (**A**) In the WM group, HCC recurrence-free survival rates at 1, 3, and 5 years for patients with AFP ≤ 400 ng/mL were 96.6%, 92.4%, and 89.4%, respectively, whereas those for patients with AFP > 400 ng/mL were 84.6%, 84.6%, and 84.6%, respectively (*p* = 0.49). (**B**) In the BM group, HCC recurrence-free survival rates at 1, 3, and 5 years for patients with AFP ≤ 400 ng/mL were 81.1%, 71.6%, and 68.4%, respectively, while those for patients with AFP > 400 ng/mL were 27.5%, 27.5%, and 27.5%, respectively (*p* < 0.05). BM, beyond the Milan criteria; HCC, hepatocellular carcinoma; WM, within the Milan criteria.

In the WM group, HCC recurrence-free survival rates at 3 and 5 years for patients with an immediate pretransplant AFP ≤ 400 ng/mL and AFP > 400 ng/mL were 92.4% and 89.4%, and 84.6% and 84.6%, respectively (*p* = 0.49). In the BM group, HCC recurrence-free survival rates at 3 and 5 years for patients with AFP ≤ 400 ng/mL were 71.6% and 68.4%, while those for patients with AFP > 400 ng/mL were 27.5% and 27.5%, respectively (*p* < 0.05) (Figure 3). The median survival time of BM patients with AFP > 400 ng/mL was 5.55 months (95% CI, 4.40–6.70). Most of BM patients with AFP > 400 ng/mL had early recurrences within 1 year.

### 3.7. Preoperative Prognostic Factors Related to Recurrence within 5 Years

As shown in Figure 2B, the RFS curve formed a plateau after about 60 months in BM patients. Therefore, we also investigated the preoperative risk factors of recurrence within 5 years after LDLT for BM patients. These results showed the preoperative factors that increased the likelihood of recurrence within 5 years following LDLT. The multivariate stepwise logistic regression model was used. We compared the pretransplant characteristics by dividing BM patients into a recurrence group (*n* = 64) and no recurrence group (*n* = 103) (Table 4). The immediate pretransplant platelet count of the recurrence group was higher than that of the no recurrence group (*p* < 0.05). The AFP > 400 ng/mL was significantly different between the two groups (*p <* 0.05). In multivariate analysis, the results showed that AFP > 400 ng/mL (OR, 7.14; 95% CI, 3.16–17.0; *p* < 0.05) and pretransplant platelet count (OR, 1.01; 95% CI, 1.00–1.02, *p* < 0.05) were independent predictors for recurrence within 5 years (Table 5).

### 3.8. Preoperative Prognostic Factors for RFS in BM Patients with Very Large HCC (≥7 cm)

**Figure 4:**
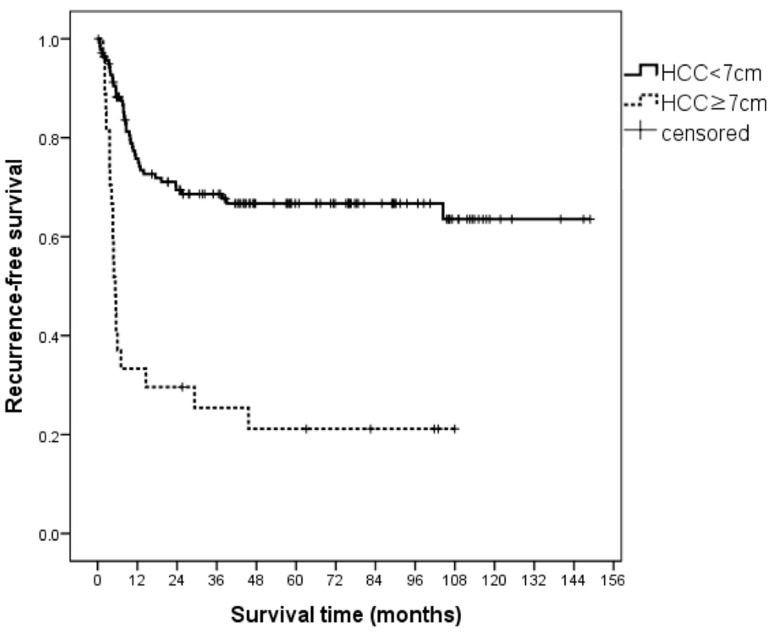
Comparisons of RFS in BM patients according to maximum tumor size (=7 cm). The 1-, 3-, and 5-year RFS of patients with HCC < 7 cm and HCC ≥ 7 cm were 75.8%, 69.6%, and 66.7%, and 33.3%, 25.4%, and 21.2% (*p* < 0.05). These patients showed a median survival time of 5.3 months (95% CI, 4.1–6.4) and 21 patients had HCC recurrence, BM, beyond the Milan criteria; HCC, hepatocellular carcinoma; RFS, recurrence-free survival.

In BM patients, we also compared the outcomes of very large HCC (≥7 cm) with smaller HCC (<7 cm). Twenty-seven (7.5%) patients had HCC ≥ 7 cm. The pathologic findings of explant liver in these patients showed a higher frequency of E–S grade III–IV, microvascular invasion, and intrahepatic metastasis than in patients with HCC < 7 cm (Table 6). The 1-, 3-, and 5-year RFS of patients with HCC < 7 cm and HCC ≥ 7 cm was 75.8%, 69.6%, and 66.7%, and 33.3%, 25.4%, and 21.2%, respectively (*p* < 0.05) (Figure 4). These patients showed a median survival time of 5.3 months (95% CI, 4.1–6.4) and 21 patients had HCC recurrence during the follow-up period. Despite the small sample size, we investigated the prognostic factors of RFS in BM patients with HCC ≥ 7 cm using pretransplant factors. In multivariate analysis, AFP > 400 ng/mL (HR, 8.23; 95% CI 2.16–31.36; *p* < 0.05) was identified as the independent risk factor for unfavorable RFS in patients with very large HCC.

## 4. Discussion

This study showed that the OS and RFS for WM patients were superior to those in the BM patients. Furthermore, the BM patients were more associated with poor histologic findings than the WM patients. In the univariate and multivariate analyses of risk factors for OS and RFS, the AFP level, the fulfillment of Milan criteria, tumor size, histologic grade, and microvascular invasion were identified as independent risk factors.

Since we initiated the LDLT program, our preferred policy for selecting recipients with HCC for LDLT has been based on the Milan criteria using pretransplant imaging tools such as liver CT, MRI, or PET/CT. In the meantime, BM patients accounted for half of the study subjects (46.5%). This is because we performed LDLT if the recipients could be expected to benefit from survival with minimal risk of surgery to the related donors, despite exceeding Milan criteria. Specifically, LDLT could be performed for BM patients in whom liver function drastically worsened, resulting in liver failure, and the related donor was willing to donate the liver. However, early recurrence and poor patient survival still place strong constraints on LDLT for BM patients. In such cases, the equipoise between the risk to a healthy donor and the benefit to a recipient with HCC should be considered [9]. Our previous study showed that all living donors recovered fully and resumed their normal activities after hepatectomy without any apparent adverse events [10]. In fact, expanded criteria for BM patients who have comparable outcomes to WM patients have also been suggested [11,12,13]. Since there is still a controversy regarding LDLT for BM patients, the implementation of LDLT should be evaluated based on survival outcomes and donor safety.

We also believe that further clarification and transparency is required with regard to the living donor’s voluntary consent to their involvement in the LDLT program. There are no mandatory guidelines for this part of the process. Of course, most living donors, such as relatives or acquaintances, feel the emotional appeal of the relationship, and we cannot persuade or stop the contribution of such donors. However, there may be other motives behind such voluntary acts. Therefore, we need the help of a social worker and psychiatrist to provide the objective judgement of a third person to evaluate the donor’s voluntary consent. They could provide counsel as to whether there are external pressures or financial factors involved and record the results as a counseling evaluation report. The evaluation report should include information such as the relationship between the recipient and the donor, any mental illness and/or intellectual disability of the donor, the motivation for donation, and the consent of the donor’s caregivers. Finally, the overall opinion can be recorded including whether the donor voluntarily decided to donate as a family member, all surrounding caregivers agree, and there was no conflict in the process. After completing this process, we can confirm the donor’s consent for donor hepatectomy. Donors are always informed that if they do not want to donate, they can quit at any time.

We confirmed that patients with HCC exceeding the Milan criteria showed poorer RFS and OS after LDLT compared to those with HCC within the criteria. These results suggest that fulfillment of the criteria is a prognostic factor for LDLT, as it is for DDLT. Furthermore, we demonstrated that HCC of BM correlated with the poor pathological features of advanced HCC. Based on the analysis of 359 explanted livers, we found that HCC of BM was associated with poor E–S histologic grade differentiation, microvascular invasion, serosal invasion, and intrahepatic metastases, which have been established as unfavorable risk factors for RFS [9,11,14]. These findings represent the significance of the Milan criteria as a surrogate for pathologic findings of HCC. Pathology of the explanted liver has been considered a prognostic factor for HCC recurrence [14,15]. Moreover, tumor size and number have been reported as critical risk factors for HCC recurrence after LT [16,17]. Because the Milan criteria were based on these morphological factors, we should preferentially consider the fulfillment of these criteria before LDLT.

AFP has been investigated for predictability of HCC recurrence because the elevation of AFP has been associated with tumor growth [18]. Previous studies have also highlighted AFP as a predictor for recurrence after LT [17,19]. Ciccarelli et al. showed that AFP was an independent risk factor for post-LT recurrence in conjunction with microvascular invasion and the number of nodules [7]. A recent study confirmed the power of AFP for prediction of tumor recurrence and found that the AFP could improve the performance of the Milan criteria [19]. In this present study, the BM patients with immediate pretransplant AFP > 400 ng/mL had worse RFS compared to those with AFP ≤ 400 ng/mL. Moreover, an immediate pretransplant AFP > 400 ng/mL in BM patients is considered a crucial factor for post-transplant recurrence within 5 years after LDLT and RFS in patients with very large HCC (≥7 cm) as well. Therefore, consideration of the Milan criteria and AFP, rather than one or the other provides a better prediction of survival outcomes of BM patients after LDLT. On the other hand, the RFS curves of the WM patients with immediate pretransplant AFP > 400 ng/mL and AFP ≤ 400 ng/mL showed no significant differences. Because most of the WM patients had an AFP ≤ 400 ng/mL, the sensitivity of the cut-off value of 400 ng/mL in detecting the statistical difference in RFS curves of WM patients might have been lowered. Although AFP > 400 ng/mL was found to be a significant, unfavorable factor for RFS in the analysis of all the patients, subgroup analysis for WM patients might be needed. However, it was not performed in this study because of the favorable OS and RFS of WM patients.

Besides the Milan criteria and AFP, the present study showed that large tumor size, E–S histologic grade III–IV, and the presence of microvascular invasion of the tumor in the explanted liver were independently associated with worse survival outcomes after LDLT, as was also observed in previous studies [3,9,16,20,21]. Most of all, tumor size has been identified as a significant prognostic factor for recurrence following LT [3,21]. We also have not recommended LDLT for patients with very large HCC (≥7 cm) due to increased risk of vascular invasion, dissemination, and poor differentiation. However, in cases where a related living donor has been eager to donate the liver graft for a family member, the LDLT was performed, even though a wretched prognosis was predicted. In this subgroup (HCC ≥ 7 cm) analyses, AFP was also identified as an important prognostic factor. Second, poorly differentiated HCC, such as an E–S histologic grade III–IV was found to be a significant risk factor associated with worse survival outcomes [20]. Third, a Japanese LDLT study group demonstrated that the presence of microvascular invasion is independently associated with increased post-transplantation recurrence [16]. Moreover, Mazzaferro et al. showed that microvascular invasion of any size and number corresponded with the deterioration of both patient survival and cumulative incidence of recurrence [21].

In order to achieve better survival following LDLT, the recipient with HCC should be preoperatively evaluated based on fulfillment of the Milan criteria and AFP. After LDLT, the pathologic findings of the explanted liver should be taken into account in the plan for follow-up and surveillance.

This study has some limitations. First, because this was a retrospective study, selection bias may be inherently present. Comorbidity, such as hypertension, diabetes mellitus, pulmonary disease, renal disease, coronary disease, or neurologic disease, was not analyzed in the study. Although some patients had mild systemic diseases, they were well-controlled and did not result in functional limitations. Second, although we performed multivariate analyses to assess the independent risk factors for survival outcomes, the sample size was small. Third, we did not check the changes in AFP levels after locoregional treatments. So, whether AFP change can also predict the prognosis of HCC patients after LDLT still needs further study. Nonetheless, the results of this study from a National Cancer Center cohort could be a useful platform on which further validation studies can be undertaken in patients with HCC undergoing LDLT.

## 5. Conclusions

The OS and RFS after LDLT for HCC of WM patients were superior to those of BM patients. Patients with HCC exceeding the Milan criteria and an immediate pretransplant AFP > 400 ng/mL had unfavorable survival outcomes following LDLT. The tumor size, poorly histologic differentiation, and the presence of microvascular invasion of HCC in the explanted liver were independent risk factors for both OS and RFS.

## Figures and Tables

**Table 1:** Baseline characteristics of patients undergoing LDLT for HCC and assessed according to the Milan criteria.

	Total (*n* = 359)	WM Patients (*n* = 192)	BM Patients (*n* = 167)	*p*
Recipient				
Male (%)	297 (82.7)	150 (78.1)	147 (88.0)	<0.05
Age (yrs)	54.2 ± 7.6	53.7 ± 7.6	54.7 ± 7.5	0.19
BMI (kg/m^2^)	23.6 (21.6–25.7)	23.8 (21.9–26.4)	23.5 (21.4–25.3)	0.07
Child-Pugh class				0.05
A	195 (54.3)	108 (56.3)	87 (52.1)	
B	98 (27.3)	43 (22.4)	55 (32.9)	
C	66 (18.4)	41 (21.3)	25 (15.0)	
MELD score	11 (6–51)	11 (6–51)	11 (6–47)	0.38
Ascites				
None	205 (57.1%)	111 (57.8%)	94 (56.3%)	0.37
Mild to moderate	110 (30.6%)	54 (28.1%)	56 (33.5%)	
Severe	44 (12.3%)	27 (14.1%)	17 (10.2%)	
GRWR	0.99 (0.42–1.93)	1.01 (0.42–1.87)	0.99 (0.45–1.93)	0.47
SFSS	84 (23.4)	42 (21.9)	42 (25.2)	0.46
ABO incompatible	34 (9.5)	20 (10.4)	14 (8.4)	0.51
Basal disease				0.76
Non-viral	23 (6.4)	13 (6.8)	10 (6.0)	
Viral	336 (93.6)	179 (93.2)	157 (94.0)	
Viral hepatitis type				0.11
B	311 (86.6)	170 (88.5)	141 (84.4)	
C	17 (4.7)	6 (3.1)	11 (6.6)	
B, C	6 (1.7)	1 (0.5)	5 (3.0)	
NBNC	25 (7.0)	15 (7.8)	10 (6.0)	
EBL (mL)	1500 (200–90,000)	1500 (300–400,000)	1500 (200–900,000)	0.33
CIT (min)	83.0 (20.0–255.0)	82.0 (20.0–255.0)	83.0 (23.0–212.0)	0.75
WIT (min)	25.0 (5.0–255.0)	26.0 (5.0–115.0)	25.0 (7.0–55.0)	0.23
Plt count (×10^3^/μL)	79.0 (15.0–399.0)	73.5 (15.0–399.0)	84.0 (20.0–286.0)	0.37
AFP (ng/mL)				<0.05
≤400	308 (85.8)	179 (93.2)	129 (77.3)	
>400	51 (14.2)	13 (6.8)	38 (22.7)	
Locoregional treatment				
Resection	59 (16.4)	29 (15.1)	30 (18.0)	0.46
TACE	225 (62.7)	107 (55.7)	118 (70.7)	<0.05
RFA	27 (7.5)	14 (7.3)	13 (7.8)	0.86
PEI	7 (2.0)	5 (2.6)	2 (1.2)	0.45
Donor				
Male	229 (63.8)	125 (65.1)	104 (62.3)	0.57
Age (yrs)	31 (16–75)	30 (16–75)	31 (16–62)	0.76
Graft type				0.48
Right liver	351 (97.8)	189 (98.4)	162 (97.0)	
Extended right liver	8 (2.2)	3 (1.6)	5 (3.0)	

Values are presented as counts with percentages for categorical data and means with standard deviations or medians with interquartile ranges for continuous data, depending on the normality of the distribution. AFP, alpha-fetoprotein; BM, beyond the Milan criteria; BMI, body mass index; CIT, cold ischemic time; EBL, estimated blood loss; GRWR, graft to recipient weight ratio; HCC, hepatocellular carcinoma; LDLT, living donor liver transplantation; LT, liver transplantation; MELD, model for end-stage liver disease; PEI, percutaneous ethanol injection; Plt, platelet; RFA, radiofrequency ablation; SFSS, small for size syndrome; TACE, transcatheter arterial chemoembolization; WIT, warm ischemic time; WM, within the Milan criteria.

**Table 2:** Comparison of pathologic findings of HCC in explanted livers according to the Milan criteria assessment.

	Total (*n* = 359)	WM Patients (*n* = 192)	BM Patients (*n* = 167)	*p*
Number (n)				<0.05
Solitary tumor	152 (42.3)	117 (60.9)	35 (21.0)	
2–3 tumors	97 (27.0)	75 (39.1)	22 (13.2)	
>3 tumors	110 (30.6)	0 (0.0)	110 (65.8)	
Maximum tumor size (cm)	3.04 ± 2.48	1.97 ± 0.85	4.26 ± 3.09	<0.05
E–S grade III–IV	105 (29.3)	44 (22.9)	61 (36.5)	<0.05
Microvascular invasion	136 (37.9)	45 (23.4)	91 (54.5)	<0.05
Bile duct invasion	12 (3.3)	4 (2.1)	8 (4.8)	0.15
Serosa invasion	77 (21.5)	23 (12.0)	54 (32.3)	<0.05
Intrahepatic metastasis	144 (40.1)	38 (19.8)	106 (63.5)	<0.05
Liver cirrhosis	334 (93.0)	181 (94.3)	153 (91.6)	0.32
Dysplasia	109 (30.4)	50 (26.0)	59 (35.3)	0.05

Values are presented as counts with percentages for categorical data and means with standard deviations or medians with interquartile ranges for continuous data, depending on the normality of the distribution. BM, beyond the Milan criteria; E–S grade, Edmondson–Steiner histologic grade; HCC, hepatocellular carcinoma; WM, within the Milan criteria.

**Table 3:** Univariate and multivariate analyses for overall and recurrence-free survivals in the total patient population.

	Total (*n* = 359)							
	Overall Survival				Recurrence-Free Survival			
	Univariate		Multivariate		Univariate		Multivariate	
	HR (95% CI)	*p*	HR (95% CI)	*p*	HR (95% CI)	*p*	HR (95% CI)	*p*
Recipient								
Male	1.92 (0.96–3.83)	0.06			1.85 (0.96–3.57)	0.06		
Age	1.00 (0.97–1.03)	0.86			0.97 (0.94–1.00)	0.05		
MELD	0.99 (0.96–1.02)	0.69			0.94 (0.90–0.98)	<0.05	0.95 (0.91–1.00)	0.08
ABO incompatible	1.48 (0.74–2.98)	0.26			1.73 (0.94–3.19)	0.07		
EBL (mL)	1.00 (1.00–1.00)	0.92			1.00 (1.00–1.00)	0.44		
CIT (min)	1.00 (0.99–1.01)	0.13			0.99 (0.99–1.00)	0.85		
WIT (min)	1.00 (0.98–1.01)	0.85			0.99 (0.98–1.01)	0.83		
Platelet count	1.00 (0.99–1.01)	0.10			1.00 (0.99–1.01)	0.13		
AFP > 400 ng/mL	4.25 (2.73–6.62)	<0.05	2.07 (1.28–3.36)	<0.05	4.62 (2.96–7.21)	<0.05	2.16 (1.34–3.49)	<0.05
Locoregional treatment	1.20 (0.76–1.89)	0.42			1.56 (0.95–2.57)	0.47		
HCC beyond the Milan	3.73 (2.33–5.97)	<0.05	2.61 (1.60–4.26)	<0.05	4.06 (2.55–6.49)	<0.05	3.01 (1.81–5.01)	<0.05
Pathologic findings								
Number (*n*)								
Solitary tumor		-				-		
2–3 tumors	0.76 (0.43–1.32)	0.33			0.47 (0.26–0.87)	0.01		
>3 tumors	1.25 (0.78–2.02)	0.34			1.20 (0.76–1.88)	0.42		
Greatest tumor size (cm)	1.22 (1.16–1.28)	<0.05	1.15 (1.07–1.23)	<0.05	1.31 (1.24–1.38)	<0.05	1.24 (1.16–1.33)	<0.05
E-S grade III–IV	3.25 (2.14–4.95)	<0.05	2.06 (1.34–3.18)	<0.05	3.76 (2.49–5.69)	<0.05	2.25 (1.45–3.49)	<0.05
Microvascular invasion	4.25 (2.72–6.64)	<0.05	2.39 (1.32–4.35)	<0.05	6.65 (4.14–10.69)	<0.05	2.15 (1.21–3.82)	<0.05
Bile duct invasion	4.02 (1.94–8.35)	<0.05	1.34 (0.60–3.01)	0.47	3.59 (1.65–7.78)	<0.05	0.98 (0.43–2.26)	0.97
Serosa invasion	3.84 (2.51–5.88)	<0.05	1.17 (0.67–2.04)	0.57	4.88 (3.22–7.38)	<0.05	1.20 (0.70–2.04)	0.49
Intrahepatic metastasis	3.04 (1.97–4.69)	<0.05	0.61 (0.31–1.20)	0.15	4.52 (2.88–7.11)	<0.05	1.41 (0.71–2.79)	0.31

Hazards ratio (HR), 95% confidence interval (CI), and *P*-values in the univariate and multivariable analyses were estimated using the Cox proportional hazards model. AFP, alpha-fetoprotein; CIT, cold ischemic time; EBL, estimated blood loss; E-S grade, Edmondson-Steiner histologic grade; MELD, model for end-stage liver disease; UV, univariate analysis WIT, warm ischemic time.

**Table 4:** Comparisons of the pretransplant characteristics in BM patients according to HCC recurrence within 5 years.

	Total (*n* = 167)	No Recurrence (*n* = 103)	Recurrence (*n* = 64)	*p*
Recipient				
Male (%)	146 (87.4)	90 (87.4)	56 (87.5)	0.99
Age (yrs)	54.8 ± 7.5	55.6 ± 6.7	53.5 ± 8.6	0.11
Child-Pugh class				0.65
A	87 (52.1)	51 (49.5)	36 (56.2)	
B	55 (32.9)	35 (34.0)	20 (31.2)	
C	25 (15.0)	17 (16.5)	8 (12.5)	
MELD score	11.0 (9.0–15.0)	12.0 (9.0–16.0)	10.0 (9.0–14.5)	0.22
Basal disease				0.40
Non-viral	10 (6.0)	6 (5.9)	4 (6.2)	
Viral	157 (94.0)	97 (94.2)	60 (93.8)	
Plt count (×10^3^/μL)	84.0 (51.5–123.5)	79.0 (46.0–105.0)	97.5 (68.5–134.5)	<0.05
AFP (ng/mL)				<0.05
≤400	129 (77.2)	92 (89.3)	37 (57.8)	
>400	38 (22.8)	11 (10.7)	27 (42.2)	
Donor				
Male (%)	104 (62.3)	67 (65.0)	37 (57.8)	0.44
Age (yrs)	31 (25–39)	31 (25–37)	31 (23.5–42)	0.74

AFP, alpha-fetoprotein; BM, beyond the Milan criteria; HCC, hepatocellular carcinoma; MELD, model for end-stage liver disease; Plt, platelet.

**Table 5:** Multivariate analysis for pretransplant predictors related to recurrence within 5 years in BM patients.

	OR	95% CI	*p*
Plt count (×10^3^/μL)	1.01	(1.00–1.02)	<0.05
AFP > 400 ng/mL	7.14	(3.16–17.0)	<0.05

Odd ratio (OR), 95% confidence interval (CI), and *p*-values in the multivariable analysis were estimated using the logistic regression model. AFP, alpha-fetoprotein; BM, beyond the Milan criteria; Plt, platelet.

**Table 6:** Comparison of pathologic findings of explant liver according to HCC maximum tumor size (=7 cm).

	Total (*n* = 359)	HCC < 7 cm (*n* = 332)	HCC ≥ 7 cm (*n* = 27)	*p*
Number (n)				<0.05
Solitary tumor	152 (42.3)	135 (40.7)	17 (63.0)	
2–3 tumors	97 (27.0)	95 (28.6)	2 (7.4)	
>3 tumors	110 (30.9)	102 (30.7)	8 (29.6)	
Maximum tumor size (cm)	3.04 ± 2.48	2.49 ± 1.31	8.94 ± 1.99	<0.05
E–S grade III–IV	105 (29.3)	91 (27.4)	14 (51.8)	<0.05
Microvascular invasion	136 (37.9)	113 (34.0)	23 (85.2)	<0.05
Bile duct invasion	12 (3.3)	10 (3.0)	2 (7.4)	0.15
Serosa invasion	77 (21.5)	57 (17.1)	20 (74.1)	<0.05
Intrahepatic metastasis	144 (40.1)	123 (37.0)	21 (77.8)	<0.05

E–S grade, Edmondson–Steiner histologic grade; HCC, hepatocellular carcinoma.

## Data Availability

The data that support the findings of this study are available from the corresponding author, S.-H.K., upon reasonable request.
